# Better utilization of inorganic nitrogen compared to organic nitrogen by a plant symbiotic fungal isolate of *Alternaria alternata*

**DOI:** 10.17912/micropub.biology.001403

**Published:** 2025-01-29

**Authors:** Beatrice Bock, Lexie Curry, Catherine Gehring

**Affiliations:** 1 Biological Sciences, Northern Arizona University, Flagstaff, Arizona, United States; 2 Center for Adaptable Western Landscapes, Northern Arizona University, Flagstaff, Arizona, United States

## Abstract

*Alternaria alternata* , a fungus that causes plant diseases, is also a Dark Septate Endophyte (DSE) that can enhance host plant growth by improving access to soil nutrients like nitrogen. To test the environmental factors influencing this relationship, we explored whether *A. alternata* can utilize both organic and inorganic nitrogen. Our results showed that an *A. alternata* isolate grew 133% larger in an inorganic nitrogen medium than in an organic nitrogen medium. These findings suggest the need for further research on other DSE taxa and nitrogen forms to better understand fungal nitrogen use.

**Figure 1. Fungal biomass gain (g) when provided with either an inorganic or organic nitrogen source:**
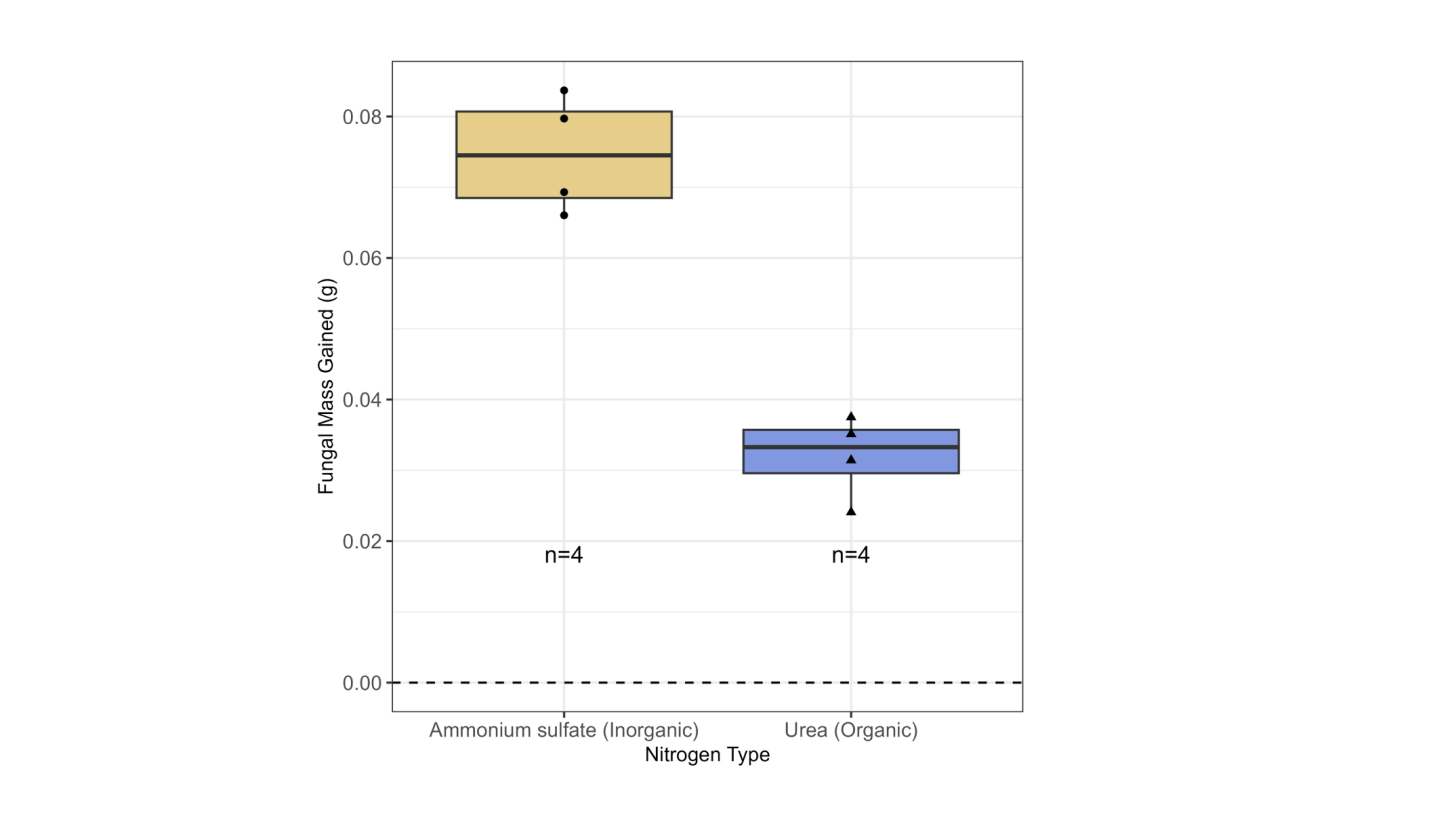
Fungal biomass was significantly higher (133%) in media containing an inorganic nitrogen source compared to an organic nitrogen source (Welch Two Sample t-test; mean _ammonium sulfate_ = 0.07468; mean _urea_ = 0.03203; t(5.3753) = 8.3487, p = 0.0003). Points are shaped according to nitrogen type and indicate each replicate in the experiment (n=4). Boxplot elements: center line, median; box limits, upper and lower quartiles; whiskers, 1.5x interquartile range; black points, observations).

## Description

*Alternaria alternata* is a fungus that causes a spot disease in many plant species, which is a concern because of the substantial declines in crop productivity that it can cause (Troncoso-Rojas & Tiznado-Hernández, 2014). *A. alternata* is classified as a Dark Septate Endophyte (DSE), which is a polyphyletic group of ascomycete fungi defined by regular cross-walls among cells (septa) and darkly pigmented hyphae (DeMers, 2022) . While some DSE are considered plant pathogens, there is increasing evidence that under certain conditions, these fungi can form positive associations with plants and improve plant growth through better access to soil resources including nitrogen (Berthelot et al., 2019; Netherway et al., 2024; Schulz & Boyle, 2005) . Given that *A. alternata* is a globally distributed species (DeMers, 2022) , it is of interest to understand the conditions that are conducive to its function as a mutualist instead of a pathogen so its management in agricultural settings can be improved. Relevant conditions affecting this relationship include the availability and amounts of different forms of nitrogen and the ratios of nitrogen to carbon available to both the plant and the fungus (Johnson, 2010) .

In this experiment, we assess the ability for an *A. alternata* isolate (GenBank Accession Number SUB14593255 germinator_fung_10x_B_2_F12 PQ284877) to utilize inorganic and organic forms of nitrogen. Nitrogen is an important nutrient for plant growth, and its use by different fungi and plants is important in agricultural settings where resources and fungal growth are monitored closely. In the context of potential nutrient-trade based symbioses between DSE and plants, it is important to know which forms of nitrogen are used and preferred by DSE and thus may be transferred to plants. Additionally, it is important to know if this fungus can use both forms of nitrogen because this will impact which forms of nitrogen are used in other experiments. For example, stable isotopes of nitrogen are useful in tracing nitrogen movement in plant-fungal symbioses. However, it is unclear which form of nitrogen is best to use for these experiments despite similar work on other groups of fungi (Finlay et al., 1992; Hawkins et al., 2000; He et al., 2003; Newsham, 2011; Upson et al., 2009).

In this laboratory experiment, we grew the *A. alternata* isolate in nitrogen-free media that was amended with either organic or inorganic nitrogen. We hypothesized that the fungus would be better able to use organic forms of nitrogen because it is a plant-symbiont that has frequent contact with plant-derived organic nitrogen (Di Martino et al., 2022; Newsham, 2011) . In contrast to our expectation, the fungal isolate’s growth was 133% higher in the inorganic nitrogen media than in the organic nitrogen media ( Figure 1 ; Welch Two Sample t-test; t(5.3753) = 8.3487, p = 0.0003). While other sources of inorganic and organic nitrogen should be tested to determine fungal utilization of the two types of nitrogen, it is clear that the fungus not only utilized an inorganic nitrogen source, but it also exhibited a statistically significant increase in fungal biomass with an inorganic nitrogen source compared to the organic nitrogen source in this experiment.

The finding that this *Alternaria sp.* DSE can utilize both inorganic and organic nitrogen sources but prefers inorganic nitrogen is helpful in thinking about its association with plants. Because DSE can act as both endophytes and as free-living saprotrophs, their preference for an inorganic nitrogen source provides both information and questions about their symbiosis with plants. If DSE can access organic nitrogen but prefer soil-derived inorganic nitrogen, then perhaps the fungus is less likely to act pathogenically upon the host plant to take its nitrogen when inorganic nitrogen is available in the rhizosphere soil surrounding a plant. In addition, if DSE prefer inorganic nitrogen sources, then perhaps their affinity for this type of nitrogen can create a nutrient gradient between the fungus and the plant, which could be part of the mechanism of how resources are traded between the two partners. In testing these ideas, these findings are relevant to a multitude of experiments, including those which aim to use stable isotopes of nitrogen to trace the movement of nitrogen, as the fungus’s ability to use the nitrogen source is vital in such a method. While more DSE isolates should be tested for their abilities to use inorganic and organic nitrogen, this experiment provides foundational evidence for DSE being able to use inorganic nitrogen sources.

## Methods

We prepared two nitrogen-free media solutions by dissolving 0.0273 grams of nitrogen-free minimal media (MyBioSource Edinburgh) per 1 mL of autoclaved RO water. These solutions were amended with either an organic or an inorganic nitrogen source. For the inorganic N treatment, we added 0.0019 grams of ammonium sulfate (Mallinckrodt Chemical Works) per 1 mL of solution. For the organic N treatment, we added 0.0009 grams of urea (Fisher Scientific U-15) per 1 mL of solution. Ismail et. al (2020) found that all 5 isolates of *Alternaria alternata* tested in their experiment produced high amounts of urease enzymes (Ismail et al., 2020) , so urea is an appropriate choice for an organic nitrogen source. Ratios were calculated based on the number of N atoms by weight to ensure the same amount of nitrogen was in each treatment. Both solutions were autoclaved and brought to room temperature over 24 hours. The pH of both liquid media was estimated using litmus paper before and after conducting this experiment, and all reads indicated neutral pH.

Four replicates were prepared for each treatment. 50 mL Erlenmeyer cell culture flasks with metal caps were autoclaved, and 15 mL of either media type was added to each flask with sterile serological pipettes in a sterile biosafety cabinet using aseptic technique. A 4 mm diameter cork borer was used to punch same-sized inoculum plugs from the growing edge of the fungal culture (GenBank Accession Number SUB14593255 germinator_fung_10x_B_2_F12 PQ284877), plated two weeks prior on Potato Dextrose Agar. The dry weight of these plugs is approximately 0.0029 g, calculated by averaging the dry weights of four inoculum plugs not used in the experiment. One fresh fungal plug was added to each flask. Flasks were incubated at 19ºC shaking at 80 RPM for 7 days. Prior to filtering the fungal contents of each flask, filter papers were labeled and their individual weights recorded. After incubation, the contents of each flask were poured through these filter papers. Flasks were rinsed with autoclaved RO water, and the contents were poured through the pre-weighed filter papers until the visible contents of all flasks were cleared. Once filtering was complete, we placed the folded filter papers upright in a rack in a 59.2ºC drying oven for 48 hours. After drying, we weighed each sample. Fungal mass was calculated by subtracting each filter paper weight and the average dry fungal plug weight from the final mass of each filtrate.

Fungal mass measurements between nitrogen treatments were compared with a Welch Two Sample t-test. We conducted all data analyses in R version 4.3.0 with RStudio (R Core Team, 2023) . R packages used include janitor for cleaning up column names in the dataset, dplyr for data processing, ggplot2 for data visualization, and EnvStats for displaying replicate numbers on the plot (Firke, 2016; Millard, 2013; Wickham et al., 2007, 2014).

## Reagents

**Table:** 

Reagent Name	Details	Available From
*Alternaria alternata* fungus	GenBank Accession Number SUB14593255 germinator_fung_10x_B_2_F12 PQ284877. Isolated from a *Sorghum bicolor* seed by Dr. Ron Deckert in Dr. Catherine Gehring’s laboratory at Northern Arizona University.	Dr. Catherine Gehring’s laboratory archive of fungi
Nitrogen free minimal media	Edinburgh minimal media, nitrogen free. Catalog #MBS652833	MyBioSource
Ammonium sulfate	Mallinckrodt Chemical Works Ammonium Sulfate	Mallinckrodt Pharmaceuticals
Urea	Fisher Scientific Urea U-15	Fisher Scientific

## References

[R1] Berthelot, C., Chalot, M., Leyval, C., & Blaudez, D. (2019). From darkness to light: Emergence of the mysterious Dark Septate Endophytes in plant growth promotion and stress alleviation. In T. R. Hodkinson, F. M. Doohan, M. J. Saunders, & B. R. Murphy (Eds.), *Endophytes for a Growing World* (1st ed., pp. 143–164). Cambridge University Press. https://www.cambridge.org/core/product/identifier/9781108607667%23CN-bp-7/type/book_part

[R2] DeMers Mara (2022). Alternaria alternata as endophyte and pathogen. Microbiology.

[R3] Di Martino Catello, Torino Valentina, Minotti Pasqualino, Pietrantonio Laura, Del Grosso Carmine, Palmieri Davide, Palumbo Giuseppe, Crawford Thomas W., Carfagna Simona (2022). Mycorrhized Wheat Plants and Nitrogen Assimilation in Coexistence and Antagonism with Spontaneous Colonization of Pathogenic and Saprophytic Fungi in a Soil of Low Fertility. Plants.

[R4] FINLAY R. D., FROSTEGÅRD Å., SONNERFELDT A.‐M. (1992). Utilization of organic and inorganic nitrogen sources by ectomycorrhizal fungi in pure culture and in symbiosis with
*Pinus contorta*
Dougl. ex Loud.. New Phytologist.

[R5] Hawkins Heidi-Jayne (2000). null. Plant and Soil.

[R6] He Xin-Hua, Critchley Christa, Bledsoe Caroline (2003). Nitrogen Transfer Within and Between Plants Through Common Mycorrhizal Networks (CMNs). Critical Reviews in Plant Sciences.

[R7] (2020). EVALUATION OF PHYSIOLOGICAL AND BIOCHEMICAL CHARACTERISTICS OF ALTERNARIA SPECIES ISOLATED FROM SOIL IN ASSIUT GOVERNORATE, EGYPT, IN ADDITION TO DICHOTOMOUS KEY TO THE ENCOUNTERED SPECIES. Assiut University Journal of Multidisciplinary Scientific Research.

[R8] Johnson, N. C. (2010). Resource stoichiometry elucidates the structure and function of arbuscular mycorrhizas across scales. *New Phytologist* , *185* (3), 631–647. https://doi.org/10.1111/j.1469-8137.2009.03110.x 10.1111/j.1469-8137.2009.03110.x 19968797

[R9] Millard, S. P. (2013). *EnvStats: Package for Environmental Statistics, Including US EPA Guidance* (p. 3.0.0) [Dataset]. https://doi.org/10.32614/CRAN.package.EnvStats

[R10] Netherway Tarquin, Bengtsson Jan, Buegger Franz, Fritscher Joachim, Oja Jane, Pritsch Karin, Hildebrand Falk, Krab Eveline J., Bahram Mohammad (2024). Pervasive associations between dark septate endophytic fungi with tree root and soil microbiomes across Europe. Nature Communications.

[R11] Newsham K. K. (2011). A meta‐analysis of plant responses to dark septate root endophytes. New Phytologist.

[R12] R Core Team. (2023). *R: A Language and Environment for Statistical Computing* [Computer software]. R Foundation for Statistical Computing. https://www.R-project.org/

[R13] Schulz Barbara, Boyle Christine (2005). The endophytic continuum. Mycological Research.

[R14] Troncoso-Rojas Rosalba, Tiznado-Hernández Martín Ernesto (2014). Alternaria alternata (Black Rot, Black Spot). Postharvest Decay.

[R15] Upson Rebecca, Read David J., Newsham Kevin K. (2009). Nitrogen form influences the response of Deschampsia antarctica to dark septate root endophytes. Mycorrhiza.

[R16] Wickham, H., Chang, W., Henry, L., Pedersen, T. L., Takahashi, K., Wilke, C., Woo, K., Yutani, H., Dunnington, D., & Van Den Brand, T. (2007). *ggplot2: Create Elegant Data Visualisations Using the Grammar of Graphics* (p. 3.5.1) [Dataset]. https://doi.org/10.32614/CRAN.package.ggplot2

[R17] Wickham, H., François, R., Henry, L., Müller, K., & Vaughan, D. (2014). *dplyr: A Grammar of Data Manipulation* (p. 1.1.4) [Dataset]. https://doi.org/10.32614/CRAN.package.dplyr

